# Supplementary material for: ARRDC4 and UBXN1: Novel Target Genes Correlated with Prostate Cancer Gleason Score
Source: Cancers (Basel). 2021 Oct 17;13(20):5209. doi: 10.3390/cancers13205209 (PMC8533922; doi:10.3390/cancers13205209)
Supplement: Supplementary file 1 [file cancers-13-05209-s001.zip › cancers-1363191supplementary.pdf]

Table S1. Primers list for real-time PCR

Gene	Forward primer 5'→3'	Reverse primer 5'→3'
UBXN1	GCCTAGGAAAAGGAGGGATG	GCCACAATGAGAACAGCAGA
ARRDC4	CCTCAACCCCCT AACTGTGA	AGGATGTGGGTCAACCTCTG
GAPDH	TGCACCACCAACTGCTTAG	AGAGGCAGGGATGATGTTC
